# Thermal properties of carbon black aqueous nanofluids for solar absorption

**DOI:** 10.1186/1556-276X-6-457

**Published:** 2011-07-18

**Authors:** Dongxiao Han, Zhaoguo Meng, Daxiong Wu, Canying Zhang, Haitao Zhu

**Affiliations:** 1 College of Materials Science and Engineering, Qingdao University of Science and Technology, Qingdao, 266042, China

**Keywords:** nanofluids, solar absorption, carbon black, photothermal properties, rheological behaviors, thermal conductivity

## Abstract

In this article, carbon black nanofluids were prepared by dispersing the pretreated carbon black powder into distilled water. The size and morphology of the nanoparticles were explored. The photothermal properties, optical properties, rheological behaviors, and thermal conductivities of the nanofluids were also investigated. The results showed that the nanofluids of high-volume fraction had better photothermal properties. Both carbon black powder and nanofluids had good absorption in the whole wavelength ranging from 200 to 2,500 nm. The nanofluids exhibited a shear thinning behavior. The shear viscosity increased with the increasing volume fraction and decreased with the increasing temperature at the same shear rate. The thermal conductivity of carbon black nanofluids increased with the increase of volume fraction and temperature. Carbon black nanofluids had good absorption ability of solar energy and can effectively enhance the solar absorption efficiency.

## Introduction

The major resource of renewable energy comes from the sun. Solar energy utilization is very important in the background of global warming and reduction of carbon dioxide emission. Solar energy has been explored through solar thermal utilization, photovoltaic power generation, and so on [1-3]. Solar thermal utilization is the most popular application among them. In conventional solar thermal collectors, plates or tubes coated with a layer of selectively absorbing material are used to absorb solar energy, and then energy is carried away by working fluids in the form of heat [4,5]. This type of collector exhibits several shortcomings, such as limitations on incident flux density and relatively high heat losses [6]. In order to overcome these drawbacks, direct solar absorption collector has been used for solar thermal utilization. In this kind of collector, solar energy is directly absorbed by the working fluids meanwhile the generated heat is carried out by the working fluids [4].

In the last century, black liquids containing millimeter to micrometer-sized particle were used as working fluid in solar collectors due to their excellent photothermal properties [7]. However, the applications of these suspensions are limited because of severe abrasion, sedimentation, and plug problems of coarse particles. Recently, nanofluids have been applied as working fluids in direct solar collectors [5,8-11]. Nanofluid is a new class of heat transfer fluids containing stably suspended nano-sized particles, fibers, or tubes in the conventional heat transfer fluids such as water, ethylene glycol, engine oil, etc. [12-16]. Several researchers have reported that nanofluids could effectively improve the solar energy utilization [4,17,18]. Taylor *et al*. found that nanofluids had excellent potential for solar thermal power plants. Efficiency improvement on the order of 5% to 10% was possible with a nanofluid receiver [19]. Shin *et al*. reported that the specific heat of a high temperature nanofluid (1 wt.% silica nanoparticles in a eutectic of lithium carbonate and potassium carbonate) enhanced by 25% compared with that of the pure eutectic [20]. The results of Tyagi *et al*. showed that the absolute efficiencies of the Al/water nanofluid-based direct absorption solar collectors were about 10% higher than that of the conventional flat-plate type collectors using pure water under similar operating conditions [6]. Mu *et al*. investigated the radiative properties of SiO_2_/water, TiO_2_/water, and ZrC/water nanofluids. They found that the ZrC nanofluid had the highest solar absorbance among the studied nanofluids [5]. However, the research on the solar energy utilization of nanofluids is only in the start stage, and the relative reports are scarce at present.

When nanofluids are used as working fluids of the direct solar absorbers, the thermal properties of nanofluids are critical to the solar utilization. Photothermal property is very important to the assessment of solar energy absorption of nanofluids because it directly reflects the solar absorption ability of nanofluids. Viscosity and rheological behaviors not only are essential parameters for nanofluid stability and flow behaviors but also affect the heat transfer efficiency of direct solar absorbers. Thermal conductivity is an important parameter for heat transfer fluids. It also affects the collectors' heat transfer efficiency. Great efforts have been made to the rheological behaviors and thermal conductivities of nanofluids [21-27], and these studies are helpful to the research of nanofluids as solar absorption working fluids. However, as mentioned above, there are only a few research committed to the photothermal properties [5,18]. Therefore, more studies are essential to the photothermal property research.

Carbon black is a kind of material that has very good absorption in the whole wavelength range of sunlight [18]. Carbon black nanofluids seem to have high potentials in the application of solar utilization. However, there are only a few researches on carbon black nanofluids [28-31], which mainly concern about the viscosity, dispersion stability, and tribological behavior.

In this study, carbon black nanofluids were prepared by dispersing the pretreated carbon black powder into distilled water. The size and morphology of the nanoparticles were explored. The photothermal properties, optical properties, rheological behaviors, and thermal conductivities of the nanofluids were also investigated.

## Experiments

### Preparation of nanofluids

Commercial carbon black powder (N115) was supplied by Qingdao Degussa Company, Qingdao, China. To obtain stable nanofluids, the original carbon black powder was pretreated as follows: 15 g of original carbon black powder and 300 ml 30% H_2_O_2 _were added into a round-bottomed flask and heated to boiling under magnetic stirring. The reaction was carried out under stirring and boiling for 5 h. Then the mixture was filtrated at room temperature and dried at 100°C. Pretreated carbon black powder was obtained by repeating the process twice. Then the pretreated carbon black powder was ground and dispersed into distilled water under ultrasonic vibration for 1 h. Carbon black nanofluids of different particle volume fractions were prepared by adjusting the amount of carbon black and water.

### Characterization of carbon black nanofluids

The transmission electron microscopy (TEM) images were captured on a JEM-2000EX (JEOL Ltd., Tokyo, Japan) transmission electron microscope with an acceleration voltage of 160 kV. The carbon black nanofluids were diluted with distilled water and one drop was placed on a carbon-coated copper grid and left to dry at room temperature. Particle size distributions of the nanoparticles in nanofluids were measured with a Zetasizer 3000HS (Malvern, Worcestershire, UK) particle size analyzer based on dynamic light scattering technology. The samples were also prepared by diluting the nanofluids with distilled water.

### Measurements of photothermal properties of carbon black nanofluids

**Figure 1:**
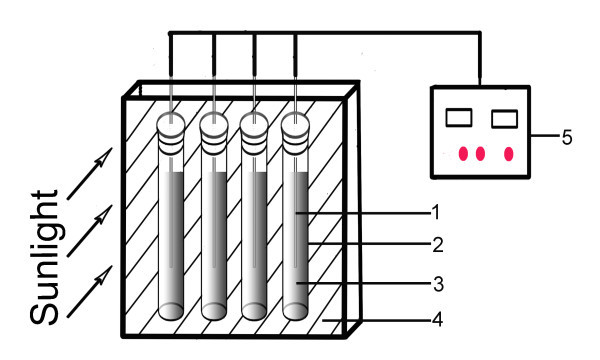
**Schematic diagram of the nanofluids photothermal property test equipment**. 1, thermocouple; 2, quartz tube; 3, nanofluids; 4, insulation materials; 5, data acquisition device.

The schematic diagram of photothermal property test equipment was shown in Figure 1. Carbon black nanofluids were sealed in quartz tubes (*d *= 26 mm, *h *= 150 mm). The tubes were placed in an insulation box. Insulation materials were put under and between the tubes. Each tube was filled with nanofluids of the same amount, so that the experimental nanofluids had the same endothermic and heat transfer area. Temperatures of the nanofluids were measured and recorded in real time with thermocouples inserted in the nanofluids. The measurements were directly carried out in the sun and performed twice and averaged. The average atmospheric temperature is 24°C.

### Measurements of optical properties of carbon black powder and nanofluids

UV-Vis-NIR spectra of pretreated carbon black powder and nanofluids were recorded on a CARY-500 spectrophotometer (MedWOW, Necosia, Cyprus) at room temperature from 200 to 2,500 nm. The carbon black powder was put on a sample stage, and the absorption spectra were detected. The carbon black nanofluids of different volume fraction were put into quartz cuvettes, and the transmittance spectra were detected.

### Measurements of rheological behaviors of carbon black nanofluids

The rheological behaviors of the carbon black nanofluids were investigated on a controlled stress viscometer (Physica MCR301, Anton Paar, Graz, Austria) with a cylindrical rotor. The shear rate and temperature ranged from 15 to 110 s^-1 ^and 25°C to 50°C, respectively. A continuous reading of shear stress and shear rate was recorded automatically when the measurement process was stabilized after the nanofluids were transferred into a measurement chamber. The cylindrical sample cell was surrounded with a constant temperature water bath. The temperature measurement accuracy was 0.01°C.

### Measurements of thermal conductivity of carbon black nanofluids

The thermal conductivity was measured on a KD2 Pro Thermal Property Analyzer (Decagon Inc., Pullman, WA, USA) using a single-needle sensor for heating and monitoring of the temperature, which is based on the transient hot wire method. The instrument's probe (1.3 mm in diameter and 60-mm long) was vertically immersed in the center of nanofluids. The thermal conductivity range of the probe was 0.02 to approximately 2 Wm^-1^K^-1^. The dimensions of cylindrical sample cell were 35 mm in diameter and 70 mm in length. Each measurement took 1 min. Calibration of the probe was carried out first by measuring the thermal conductivity of pure water, ethylene glycol, and glycerol. All our measurements were performed over ten times and averaged, and the time interval between the measurements was 15 min.

## Results and discussion

### Characterization of typical sample

**Figure 2:**
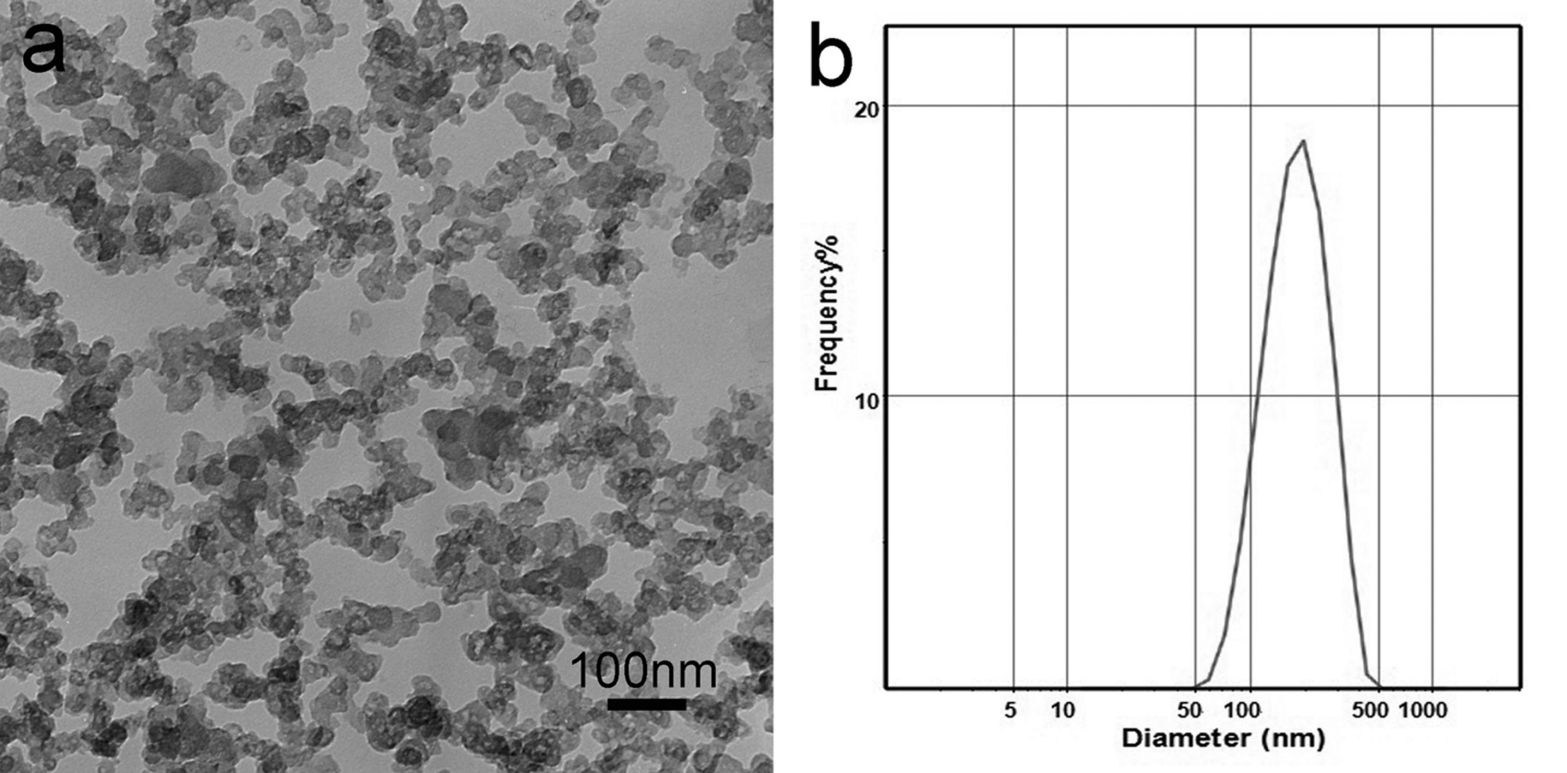
**Characterization of the typical sample**. **(a) **TEM image, **(b) **size distributions.

Figure 2a shows the TEM image of the carbon black nanofluids. The primary nanoparticles are about 20 nm in diameter and aggregate to short clusters. Figure 2b shows the size distributions of carbon black nanofluids. The particle size of the carbon black nanofluid is about 50 to 500 nm and has a mean size of 190 nm. The agglomeration of the nanoparticles and the hydrodynamic diameter measured by the Malvern particle size analyzer are responsible for the larger particle size [21].

### Photothermal properties of carbon black nanofluids

**Figure 3:**
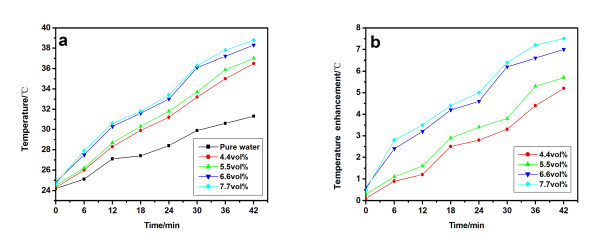
**Photothermal properties of carbon black nanofluids**. **(a) **Temperature as a function of time, **(b) **temperature enhancement as a function of time.

Figure 3a shows the temperatures of carbon black nanofluids and pure water as a function of the solar irradiation time. Figure 3b shows the temperature enhancement of nanofluids to pure water at the same irradiation time. It can be seen that the temperatures of the nanofluids increase more quickly than that of pure water. For example, within 42 min, the temperature of the 6.6 vol.% nanofluid increases from 24.4°C to 38.4°C while that of the pure water only increases to 31.2°C (Figure 3a). This indicates that carbon black nanofluids have good solar energy adsorption properties. It is clear that the nanofluids of high-volume fraction show higher temperatures, i.e., the solar adsorption ability enhances with the volume fraction in the experimental range (Figure 3). However, the temperature of 7.7 vol.% nanofluids is close to that of 6.6 vol.% sample, indicating that the photothermal properties will not change significantly when the volume fraction is higher than 6.6 vol.%. The temperature enhancements of carbon black nanofluids were higher than that of Mu's TiO_2_/water, SiO_2_/water, and ZrC/water nanofluids (<1 wt.%) [5], it is maybe due to the high concentration and good solar absorption of carbon black nanofluids (see the following section).

### Optical properties of carbon black powder and nanofluids

**Figure 4:**
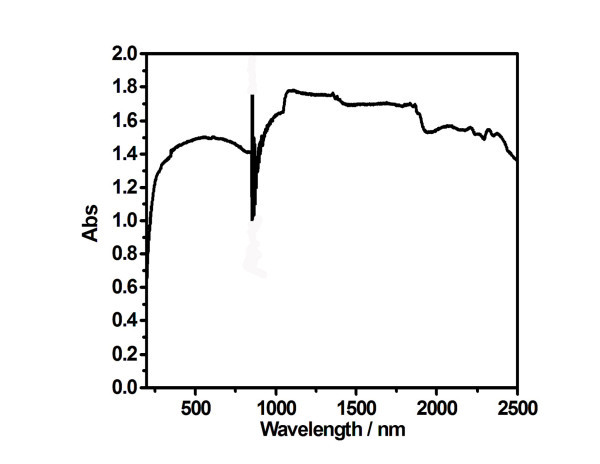
**UV-Vis-NIR absorption spectra of carbon black powder**.

Figure 4 shows the UV-Vis-NIR absorption spectra of carbon black powder. The fluctuations from 800 to 950 nm are due to wave change of the equipment. It is clear that carbon black powder has very good absorption in the whole range.

**Figure 5:**
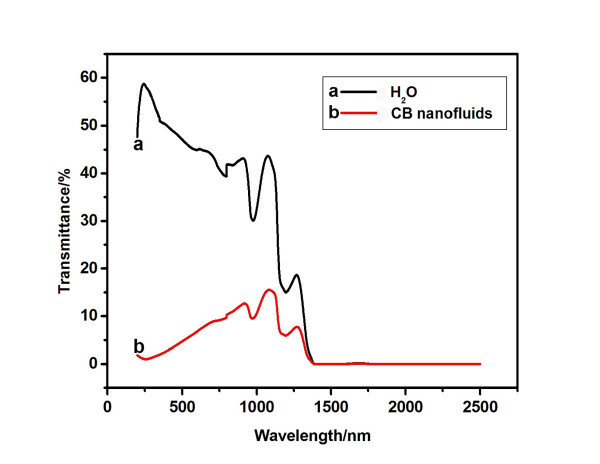
**UV-Vis-NIR transmittance spectra**. **(a) **Water, **(b) **carbon black nanofluids.

Figure 5 shows the UV-Vis-NIR transmittance spectra of water and carbon black nanofluids. It can be seen that both the water and carbon black nanofluids have perfect absorption in the wavelength ranging from 1,400 to 2,500 nm, and carbon black nanofluids have lower transmittance than water in the wavelength ranging from 200 to 1,400 nm, indicating better solar absorption ability. These are responsible for the better photothermal properties of carbon black nanofluids.

### Rheological behaviors of carbon black nanofluids

**Figure 6:**
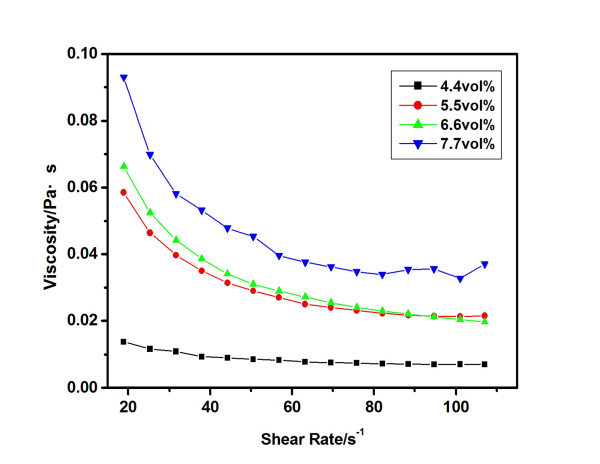
**Rheological behaviors of carbon black nanofluids of different concentrations at 27°C**.

Figure 6 shows the rheological behaviors of carbon black nanofluids for different concentrations at room temperature (27°C). A shear thinning behavior can be observed, and the extent of the shear thinning behavior increases with the carbon black concentration. The shear viscosity also increases with the increasing carbon black concentration at the same shear rate. The shear thinning behavior of present nanofluids is maybe due to the high concentration and aggregation structure of nanoparticles. It agrees with the results of Tseng *et al*. for concentrated (5 to approximately 12 vol.%) aqueous suspensions of TiO_2 _[32] and that of Tamjid *et al*. for Ag/diethylene glycol (0.2 to approximately 4.37 vol.%) [33].

As the shear rate increases, the aggregation structures of the nanoparticles break down. As a result, the viscosity decreases, and shear thinning behaviors are observed. With the increase of the carbon black concentration, the interaction between the nanoparticles enhances, and the flow resisting force increases. Therefore, the viscosity and the heat resistance increase with the increase of the volume fraction.

**Figure 7:**
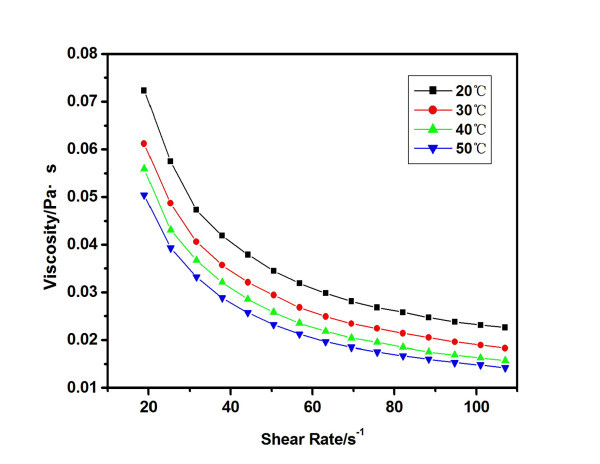
**Rheological behaviors of carbon black nanofluids (6.6 vol.%) at different temperatures**.

Figure 7 shows the rheological behaviors at different temperatures for the 6.6 vol.% carbon black nanofluids. The nanofluids at other concentrations have the similar rheological behavior. A shear thinning behavior can be observed obviously, and the shear viscosity decreases with the increase of the temperature at the same shear rate.

With the increase of the temperature, Brownian motion enhances, and hence, the interaction between the nanoparticles decreases. The solvent effect of the carbon black particles also decreases at high temperatures. These might be responsible for the small viscosity at high temperatures. When the temperature goes up, the viscosity of the nanofluids decreases, and thus the flow resisting force and heat resistance decreases. This is helpful to improve the efficiency of the solar absorbers at high temperatures.

### Thermal conductivity of carbon black nanofluids

**Figure 8:**
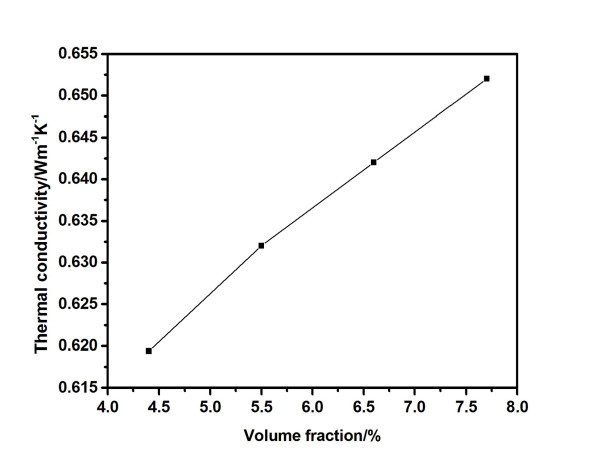
**Thermal conductivity of nanofluids as a function of carbon black volume fraction at 35°C**.

Figure 8 shows the thermal conductivity of carbon black nanofluids for different concentrations at 35°C. The nanofluids at other temperatures (ranging from 15°C to 55°C) have the similar trends. It can be seen that the thermal conductivity of nanofluids increases with the increase of carbon black volume fraction. For example, the thermal conductivities of current nanofluids are 0.619, 0.632, 0.643, and 0.652 Wm^-1^K^-1^, correlated to volume fractions of 4.4%, 5.5%, 6.6%, and 7.7%, respectively. The experimental data show a near linear correlation between the thermal conductivity and the volume fraction of carbon black. It agrees with the results in the literatures [34-36]. The thermal conductivity enhancements of current nanofluids are smaller than the reported results of functionalized carbon black nanofluids [30], which can probably be attributed to the surface functionalization of carbon black nanoparticles.

When the volume fraction increases, the effective medium increases. As a result, the thermal conductivity increases with the volume fraction. As mentioned above, the solar adsorption ability also enhances with the volume fraction. However, as the concentration of carbon black increases, the viscosity and flow resisting force increases. Thus, the heat transfer efficiency decreases. Therefore, there should be an optimum volume fraction. Considering these thermal properties, the 6.6 vol.% carbon black nanofluids have better solar thermal utilization properties.

**Figure 9:**
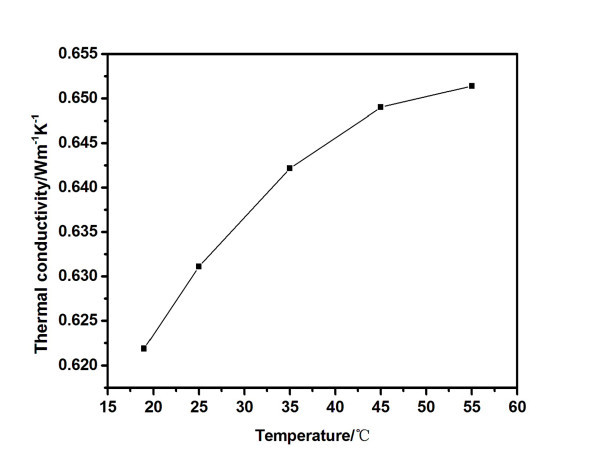
**Thermal conductivity of 6.6 vol.% carbon black nanofluids as a function of temperature**.

The thermal conductivity of carbon black nanofluids at the concentration of 6.6 vol.% is shown in Figure 9. The nanofluids at other concentrations have the similar trends. The thermal conductivity increases with the increasing temperature. For example, the thermal conductivity of nanofluid increases from 0.622 to 0.652 Wm^-1^K^-1 ^when the temperature increases from 18.5°C to 55°C. The same trend had been observed by other researchers [37-40]. The carbon black nanofluid shows high thermal conductivities at high temperatures. This can effectively improve the solar energy utilization at high temperatures. At the same time, the viscosity decreases with the increase of temperature. Therefore, the carbon black nanofluids had better solar absorption properties at higher temperatures.

## Conclusion

Carbon black nanofluids were prepared by dispersing the pretreated carbon black powder into distilled water. The nanofluids of high-volume fraction have better photothermal properties which indicate better solar energy adsorption properties. Both carbon black powder and nanofluids have good absorption in the whole wavelength range from 200 to 2,500 nm. The nanofluids exhibit a shear thinning behavior. The shear viscosity increases with the increasing volume fraction and decreases with the increasing temperature at the same shear rate. The thermal conductivity of carbon black nanofluids increases with the increase of volume fraction and temperature. In conclusion, carbon black nanofluids have good absorption ability of solar energy and can effectively enhance the solar absorption efficiency. As a result, carbon black nanofluids have high potentials for the application of solar utilization.

## Abbreviations

TEM: transmission electron microscopy.

## Competing interests

The authors declare that they have no competing interests.

## Authors' contributions

DH conducted the experiments and drafted the manuscript. ZM, DW, and CZ participated in the design of the study and revised the manuscript. HZ designed and led the work.
